# A Predictive Model for Surgical Approach Selection in Robotic Partial Nephrectomy and Its Perioperative Outcomes Based on Single‐Center Retrospective Data

**DOI:** 10.1002/cam4.70625

**Published:** 2025-02-11

**Authors:** Fan Shu, Zhuo Liu, Peichen Duan, Yichang Hao, Xin Ma, Hongxian Zhang, Guoliang Wang, Xiaojun Tian, Lei Liu, Shudong Zhang

**Affiliations:** ^1^ Department of Urology Peking University Third Hospital Beijing People's Republic of China; ^2^ Department of Urology Tongxin People's Hospital, Tongxin Ningxia People's Republic of China

**Keywords:** prediction model, propensity score matching, retroperitoneal approach, robotic partial nephrectomy, transperitoneal approach

## Abstract

**Objectives:**

To compare the perioperative and postoperative outcomes of transperitoneal and retroperitoneal robotic partial nephrectomy (RPN) and develop a prediction model for reference to select the approach.

**Materials and Methods:**

We retrospectively reviewed our single‐institutional RPN database. The patients were divided into training and validation sets. In training set, transperitoneal and retroperitoneal cases were matched using propensity score matching to balance confounding factors. The intraoperative and postoperative outcomes of both approaches were compared. A prediction model was constructed to predict the probability of the retroperitoneal approach. The model was then externally validated using the data from the validation set.

**Results:**

A total of 318 patients were included in the training set, and after propensity score matching, 200 cases were left. Additionally, 92 patients were included in the validation set. The estimated blood loss (*p* = 0.021) and the hemoglobin change (*p* = 0.016) were greater in the transperitoneal group. There was no significant difference in operative time (*p* = 0.539), warm ischemia time (*p* = 0.678), hospitalization time (*p* = 0.673), extubation time (*p* = 0.621), creatinine change (*p* = 0.623), negative margin (*p* = 1), local recurrence (*p* = 1), postoperative complication (*p* = 0.229), long‐term creatinine (*p* = 0.158), and overall survival (*p* = 0.671) between the two groups. Tumor diameter, anteroposterior location, longitudinal location, and accessory renal artery were employed as variables to construct the prediction model, resulting in area under the curve values of 0.84 and 0.77, respectively, during internal and external validation.

**Conclusions:**

Retroperitoneal and transperitoneal approaches of RPN showed no difference in perioperative outcomes except estimated blood loss and hemoglobin change. The retroperitoneal approach is recommended for smaller tumors located in the upper pole or posterior and the presence of an accessory renal artery. Our model is available to predict the probability of the retroperitoneal approach.

## 1 Introduction

Renal cell carcinoma (RCC) accounts for 2%–3% of adult malignancies and is a prevalent malignant tumor of the urinary system [1, 2]. Recently, more and more RCC are discovered in the early stages, particularly cT1 stage tumors [3]. The standard treatment for small renal masses was partial nephrectomy (PN) [3, 4]. PN has switched from an open to a minimally invasive technique due to decreased complication rates, less blood loss, shorter hospitalization time [5, 6]. The Da Vinci robotic system improves surgical efficiency and reduces difficulty, leading to its gradual adoption by urologists [7].

The retroperitoneal robotic partial nephrectomy (RRPN) and the transperitoneal robotic partial nephrectomy (TRPN) are the two surgical approaches available for robotic partial nephrectomy (RPN) [8]. Several earlier studies have compared the difficulty of operation and patients' prognosis between these two approaches [4, 7, 9, 10, 11]. Each of them has unique benefits and drawbacks as well as various application scenarios. When a surgeon is faced with an arbitrary RCC patient, how to select the best surgical approach in light of the preoperative information and the patient's condition is a conundrum that has not been satisfactorily resolved in clinical practice [12]. The majority of surgical judgments are dependent on the surgeon's personal preference and subjective experience because there is currently no quantitative tool to assist surgeons in making a rapid and accurate decision.

The current study relied on a single‐institutional retrospective database of TRPN and RRPN, to perform a comparison of perioperative indicators between the two approaches. The objective is to identify the factors associated with the surgical approach and develop a predictive model that maybe potentially aid surgeons (especially young surgeons with less experience) in making clinical decisions. On the other hand, although numerous studies have compared the differences in perioperative and postoperative outcomes of different surgical approaches [4, 7, 9, 11, 13, 14], we would like to report the data from our center. The main objective of this study is to exploratively develop a preliminary prediction model based on existing data to quantify the key factors influencing the choice of surgical approach and to investigate the impact of the surgical approach on postoperative recovery. This study is intended to lay the foundation for larger prospective studies or randomized controlled trials in the future.

## 2 Materials and Methods

### 2.1 Patients

**FIGURE 1:**
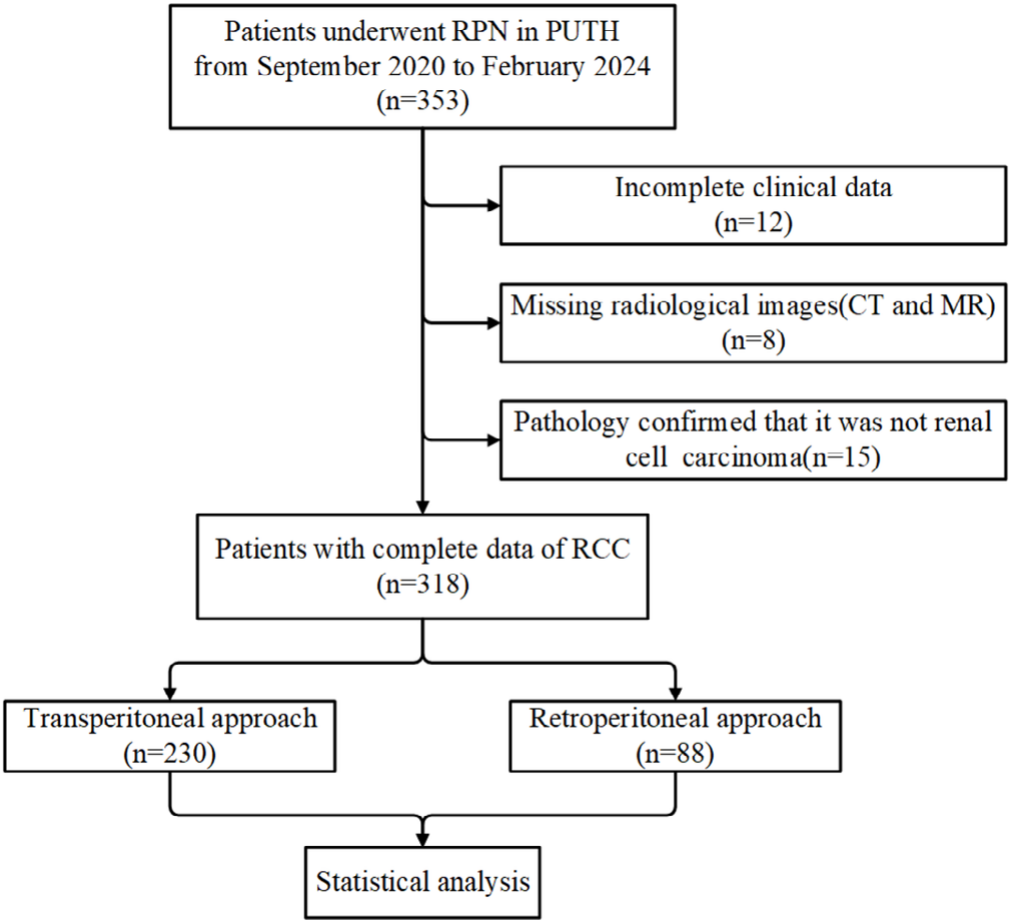
Flow chart of the study cohort. CT, computerized tomography; MR, magnetic resonance; PUTH, Peking University Third Hospital; RAPN, robotic partial nephrectomy; RCC, renal cell carcinoma.

Patients who underwent RPN at Peking University Third Hospital between September 2020 and December 2024 were retrospectively collected. Eight senior and experienced urologists completed the operations. Cases with inadequate clinical or imaging data and postoperative pathological confirmation that the primary tumor was not RCC (such as urothelial carcinoma, angiomyolipoma, etc.) were excluded. A total of 455 patients were examined and divided into a training set and a validation set based on the cutoff date of February 29, 2024. After excluding patients who did not meet the criteria, 410 patients were included for subsequent analysis (Figure 1).

### 2.2 Data Collection

Preoperative variables comprised two aspects: demographics and oncology characteristics. The former encompassed gender, age, height, weight, hypertension, diabetes, surgical history, creatinine, hemoglobin, and the American Society of Anesthesiologists (ASA) grade. The latter included tumor size, side, anteroposterior location, longitudinal location, the presence of accessory renal artery, R. E. N. A. L. score, PADUA score, mayo adhesive probability (MAP) score, and “3S + f” score. Intraoperative variables included operative time, renal artery clamping, estimated blood loss (EBL), and warm ischemic time (WIT). Postoperative measures comprised clinical and pathological data. The clinical outcomes took into account hospitalization time, extubation time, complications, and changes in creatinine and hemoglobin levels. Pathological features involved pathological type, WHO/ISUP grade, renal capsule invasion, vascular invasion, and surgical margin. The four aforementioned nephrometry scores were assessed according to the computerized tomography (CT) or magnetic resonance (MR) images of the patient. We follow patients through medical records via follow‐up visits and telephone calls to determine their survival status, whether local tumor recurrence has occurred, and creatinine levels 6–12 months after surgery. Overall survival (OS) was calculated from the date of surgery until the date of death or last follow‐up.

### 2.3 Surgical Technique

All RPN procedures were performed using the Multi‐Port Da Vinci Xi robot. All procedures were performed by eight experienced surgeons who had completed the learning curve for RPN, ensuring their competence and proficiency in performing the surgery safely and effectively.

TRPN is conducted with the patient positioned in a lateral decubitus position at 60°–70° with a waist pillow for optimal flank extension. A longitudinal skin incision of approximately 12 mm is made 2 cm above the umbilicus to place the robotic laparoscope. The trocar is then directly inserted and pneumoperitoneum pressure is maintained at 12 mm Hg following lens insertion to confirm the puncture into the abdominal cavity. Then, the additional ports were placed under direct vision. Surgery was performed with mobilization of the colon and subsequent preparation of the renal hilum.

RRPN is performed with the patient positioned in a lateral decubitus position while the operating table is fully flexed to maximize flank extension. An incision is made above the iliac crest in the mid‐axillary line, and the oblique muscles are dissected using a finger. A balloon‐dilating device is then inserted into the space outside of Gerota's fascia and inflated with 700 mL of air. Following this, a 0° robotic laparoscope is placed through the Hasson trocar. Following direct vision, the additional ports were placed. After docking, the psoas muscle was identified. Gerota's fascia was incised and the renal artery was dissected.

For both approaches, the renal fat sac around the tumor was dissected to clearly show the tumor and its surrounding tissues. The non‐injury vascular occlusion clip was inserted by the assistant from the auxiliary hole, the renal artery was blocked, and the timing started. The tumor was completely removed approximately 0.5 cm from the edge of the tumor, and then the wound was sutured. Finally, the blockade of the renal hilum was released.

### 2.4 Statistical Analysis

Statistical analysis was performed using R software (version 4.2.1) and GraphPad Prism (version 10.1.2). Normally distributed continuous variables were described using the mean and standard deviation, and statistical analysis was performed using t‐tests. Non‐normally distributed continuous variables were described through the use of median and interquartile spacing, with the Wilcoxon rank sum test employed to confirm differences. For categorical variables, Fisher's exact test and Pearson's chi‐squared test were used. Propensity score matching (PSM) was conducted to balance confounding factors between the two groups. Logistic regression analysis was employed to identify the factors linked to the surgical approach. The screening of variables in multivariate analysis was based on the results of univariate analysis and clinical experience. Kaplan–Meier survival analysis was utilized to plot survival curves. All tests were two‐tailed, with statistical significance being defined as *p* < 0.05.

## 3 Results

### 3.1 Baseline Characteristics of the Patients in the Training Set

Preoperative baseline characteristics of patients are summarized in Table 1. Of the 318 patients who were included as the training group, 72.3% (230) received TRPN, while 27.7% (88) received RRPN. The retroperitoneal and transperitoneal groups showed significant differences in gender, preoperative creatinine, and ASA grade within the unmatched cohort. After a PSM using the greedy nearest‐neighbor algorithm with a caliper value of 0.05 and a matching ratio of 1:2, 87.5% (*n* = 77) of retroperitoneal cases and 123 cases in transperitoneal groups were matched, and all the demographic data showed no significant differences.

**TABLE 1:** Comparison of baseline characteristics between transperitoneal and retroperitoneal approaches before and after PSM.

Variable	Before PSM			After PSM		
	TRPN (*n* = 230)	RRPN (*n* = 88)	*p*	TRPN (*n* = 123)	RRPN (*n* = 77)	*p*
Age, years (mean [SD])	54.78 [12.87]	53.05 [12.64]	0.281	53.45 [13.19]	53.48 [12.82]	0.986
Weight, kg (mean [SD])	74.01 [12.29]	74.31 [12.60]	0.843	73.55 [13.20]	73.69 [12.89]	0.94
Hight, cm (mean [SD])	169.78 [7.77]	169.73 [7.81]	0.955	169.20 [8.27]	169.34 [7.99]	0.91
HGB, g/L (median [IQR])	151.00 [139.00, 159.00]	148.00 [135.00, 157.00]	0.248	150.00 [137.50, 159.00]	148.00 [135.00, 157.00]	0.447
CR, μmol/L (median [IQR])	83.00 [70.00, 92.75]	78.00 [66.75, 86.25]	0.021	80.00 [67.00, 88.00]	77.00 [67.00, 88.00]	0.601
Gender, *n* [%]						
Male	174 [75.7]	56 [63.6]	0.045	83 [67.5]	51 [66.2]	0.978
Female	56 [24.3]	32 [36.4]		40 [32.5]	26 [33.8]	
Hypertension, *n* [%]						
No	149 [64.8]	51 [58.0]	0.318	72 [58.5]	46 [59.7]	0.983
Yes	81 [35.2]	37 [42.0]		51 [41.5]	31 [40.3]	
Diabetes, *n* [%]						
No	195 [84.8]	74 [84.1]	1	101 [82.1]	66 [85.7]	0.637
Yes	35 [15.2]	14 [15.9]		22 [17.9]	11 [14.3]	
Surgical history, *n* [%]						
No	141 [61.3]	63 [71.6]	0.114	87 [70.7]	52 [67.5]	0.749
Yes	89 [38.7]	25 [28.4]		36 [29.3]	25 [32.5]	
ASA grade, *n* [%]						
1	56 [24.3]	34 [38.6]	0.033	35 [28.5]	29 [37.7]	0.156
2	160 [69.6]	48 [54.5]		83 [67.5]	42 [54.5]	
3	14 [6.1]	6 [6.8]		5 [4.1]	6 [7.8]	

Abbreviations: CR, creatinine; HGB, hemoglobin; IQR, interquartile range; PSM, propensity score matching; RRPN, retroperitoneal robotic partial nephrectomy; SD, standard deviation; TRPN, transperitoneal robotic partial nephrectomy.

### 3.2 Differences in Intra‐ and Postoperative Indicators Between the Two Approaches After PSM

**FIGURE 2:**
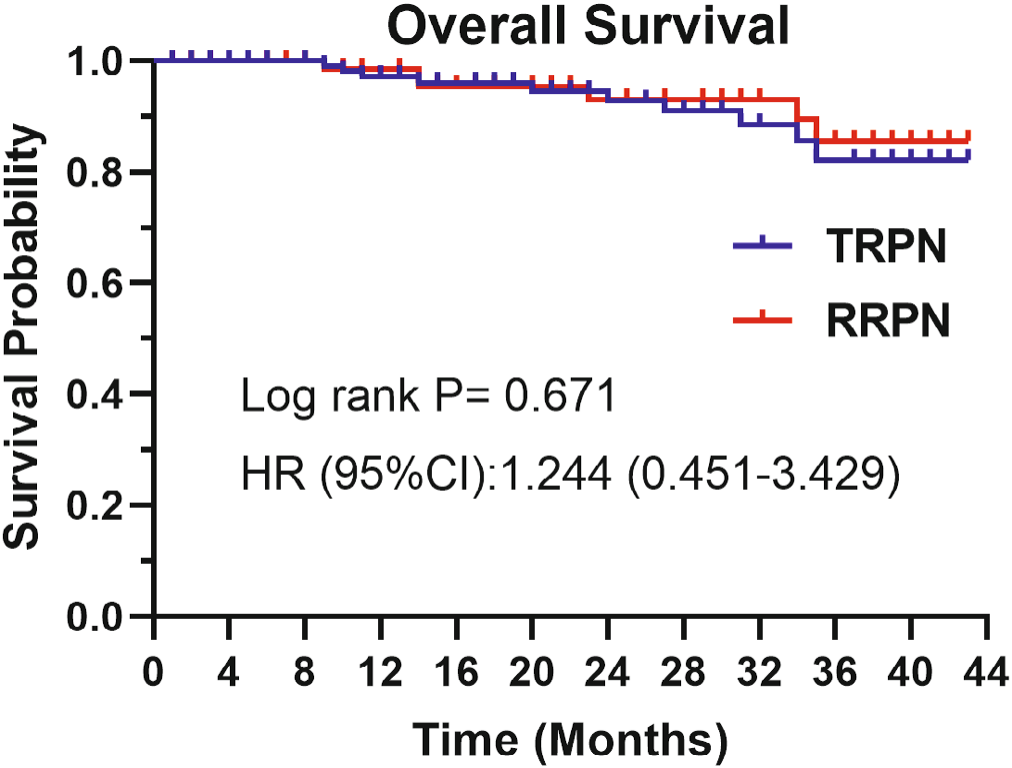
Survival curves of patients undergoing retroperitoneal and transperitoneal approaches. HR, hazard ratio; RRPN, retroperitoneal robotic partial nephrectomy; TRPN, transperitoneal robotic partial nephrectomy.

Table 2 displays the differences in perioperative outcomes between the two groups after PSM. No significant difference was observed in operative time and WIT in TRPN vs. RRPN (120.00 min vs. 117.00 min, *p* = 0.539; 17.41 min vs. 17.92 min, *p* = 0.678, respectively). The mean hospitalization time was about 5 days in both groups and no statistic difference (5.07 days vs. 5.18 days, *p* = 0.673). The EBL (40.00 mL vs. 20.00 mL, *p* = 0.021) and hemoglobin change (−20.00 g/L vs. −15.00 g/L, *p* = 0.016) was higher in the TRPN. The postoperative creatinine did not change significantly compared with preoperative in both groups. Histopathology revealed that clear cell renal cell carcinoma (ccRCC) was the most common entity (81.3% vs. 84.4%, *p* = 0.709), and there were no significant differences in surgical margin, long‐term creatinine (6–12 month after surgery), local recurrence rate, nuclear grade, and capsule and vascular invasion. In the postoperative course, complications were documented in 16 patients, with 8.9% in the TRPN group and 6.5% in the RRPN group (*p* = 0.368). During the median follow‐up periods of 24 and 21 months, respectively, the OS of patients in the RRPN and TRPN groups showed no statistically significant difference (Figure 2).

**TABLE 2:** Intra‐ and postoperative results after PSM of transperitoneal and retroperitoneal approaches.

Variable	TRPN (*n* = 123)	RRPN (*n* = 77)	*p*
Operative time, min (median [IQR])	120.00 [95.00, 157.50]	117.00 [96.00, 154.00]	0.539
EBL, mL (median [IQR])	40.00 [20.00, 150.00]	20.00 [10.00, 50.00]	0.021
WIT, min (mean [SD])	17.41 [8.48]	17.92 [8.59]	0.678
HGB change, g/L (median [IQR])	−20.00 [−26.00, −9.00]	−15.00 [−22.00, −2.00]	0.016
CR change, μmol/L (median [IQR])	1.00 [−7.00, 11.00]	0.00 [−9.00, 10.00]	0.623
Hospitalization time, d (mean [SD])	5.07 [2.13]	5.18 [1.48]	0.673
Extubation time, d (mean [SD])	3.90 [2.80]	4.09 [2.31]	0.621
Long term creatinine, μmol/L (median [IQR])	84.00 [69.25, 95.75]	80.00 [71.00, 89.00]	0.158
Follow‐up, months (median [IQR])	21.00 [13.00, 34.00]	24.00 [15.50, 36.50]	0.137
Renal artery clamping, *n* [%]			
Yes	118 [95.9]	76 [98.7]	0.49
No	5 [4.1]	1 [1.3]	
Pathological type, *n* [%]			
ccRCC	100 [81.3]	65 [84.4]	0.709
nccRCC	23 [18.7]	12 [15.6]	
WHO/ISUP grade, *n* [%]			
1	25 [20.3]	10 [13.0]	0.341
2	83 [67.5]	59 [76.6]	
3	15 [12.2]	8 [10.4]	
Renal capsule invasion, *n* [%]			
No	112 [91.1]	70 [90.9]	1
Yes	11 [8.9]	7 [9.1]	
Vascular invasion, *n* [%]			
No	119 [96.7]	75 [97.4]	1
Yes	4 [3.3]	2 [2.6]	
Complication, *n* [%]			
No	112 [91.1]	72 [93.5]	0.368
Yes	11 [8.9]	5 [6.5]	
Surgical margin, *n* [%]			
Negative	120 [97.6]	75 [97.4]	1
Positive suspiciously^a^	3 [2.4]	2 [2.6]	
Local recurrence, *n* [%]			
No	118 [95.9]	74 [96.1]	1
Yes	5 [4.1]	3 [3.9]	

Abbreviations: CR, creatinine; EBL, estimated blood loss; HGB, hemoglobin; IQR, interquartile range; RRPN, retroperitoneal robotic partial nephrectomy; SD, standard deviation; TRPN, transperitoneal robotic partial nephrectomy; WIT, warm ischemic time.

^a^ Positive suspiciously: Cancer cells and suspicious but not certain cancer cells can be seen at the pathological specimen of margins.

### 3.3 Prediction Model Based on Logistic Regression

**FIGURE 3:**
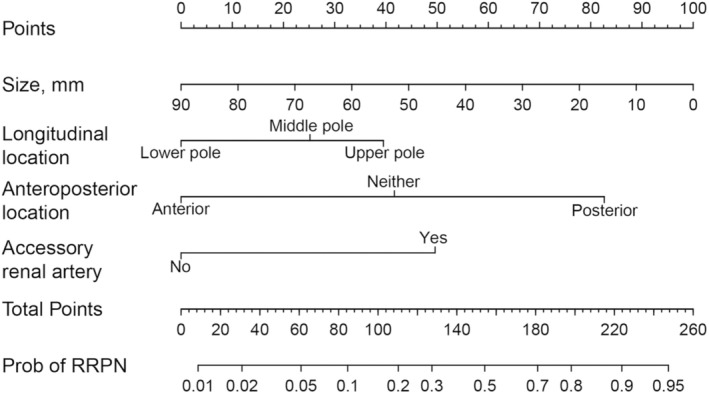
Preoperative nomogram for predicting the probability of recommending a retroperitoneal approach. The preoperative variables are presented in rows two to five. The first step is to compute points by drawing a vertical line from each variable axis upward to the point's axis. Then sum up the four points and draw a vertical line from the total points line downward to the last row to work out the probability of retroperitoneal approach.

Logistic regression analysis was conducted using the transperitoneal approach as the reference and the retroperitoneal approach as the predictor variable. Results from both univariate and multivariate analyses are presented in Table 3. In univariate analysis, tumor diameter, anteroposterior location, longitudinal location, and accessory renal artery were statistically significant. Finally, the above four factors related to the retroperitoneal approach were included, and the logistic regression equation is $\ln\left(\frac{p}{1-p}\right)=-1.71-0.04\times \text{Size}+L+M+N$, where p is the probability of the retroperitoneal approach. The unit of size is mm; L was 1.57 when accessory renal artery was present and 0 otherwise; M was 0, 1.32, and 2.61 when the tumor located in anterior, neither, and posterior, respectively; and N was 0, 0.79, and 1.25 when the tumor located in lower, middle, and upper poles, respectively. A nomogram was further drawn to facilitate the visualization of the model, as shown in Figure 3.

**TABLE 3:** Univariable and multivariable logistic regression analyses of the influence factor for the retroperitoneal approach.

Variables	Univariable logistic regression		Multivariable logistic regression	
	OR (95% CI)	*p*	OR (95% CI)	*p*
Age (years)	1.00 (0.98–1.02)	0.986	—	—
Weight (kg)	1.00 (0.98–1.02)	0.939	—	—
Hight (cm)	1.00 (0.97–1.04)	0.909	—	—
HGB (g/L)	1.00 (0.98–1.01)	0.919	—	—
CR (μmol/L)	1.00 (0.98–1.01)	0.794	—	—
Tumor size (mm)	0.96 (0.94–0.99)	0.006	0.97 (0.93–0.99)	0.037
Gender				
Male	1.00 (Reference)	—	—	—
Female	1.06 (0.58–1.94)	0.855	—	—
Hypertension				
No	1.00 (Reference)	—	—	—
Yes	0.95 (0.53–1.70)	0.866	—	—
Diabetes				
No	1.00 (Reference)	—	—	—
Yes	0.77 (0.35–1.68)	0.505	—	—
Accessory renal artery				
No	1.00 (Reference)	—	1.00 (Reference)	—
Yes	3.94 (1.82–8.51)	< 0.001	4.79 (1.89–12.16)	< 0.001
Surgical history				
No	1.00 (Reference)	—	—	—
Yes	1.16 (0.63–2.15)	0.633	—	—
Tumor side				
Left	1.00 (Reference)	—	—	—
Right	0.79 (0.45–1.41)	0.428	—	—
Anteroposterior location				
Anterior	1.00 (Reference)	—	1.00 (Reference)	—
Neither	2.78 (1.21–6.39)	0.016	3.72 (1.43–9.67)	0.007
Posterior	11.07 (5.22–23.48)	< 0.001	13.59 (5.84–31.59)	< 0.001
Longitudinal location				
Lower pole	1.00 (Reference)	—	1.00 (Reference)	—
Middle pole	2.51 (1.15–5.48)	0.021	2.21 (0.87–5.61)	0.094
Upper pole	3.19 (1.48–6.88)	0.003	3.48 (1.36–8.89)	0.009
ASA grade				
1	1.00 (Reference)	—	—	—
2	0.61 (0.33–1.13)	0.117	—	—
3	1.45 (0.40–5.23)	0.572	—	—
R.E.N.A.L. score				
4–6	1.00 (Reference)	—	—	—
7–9	1.31 (0.72–2.40)	0.374	—	—
10–12	0.83 (0.26–2.62)	0.748	—	—
PADUA score				
4–6	1.00 (Reference)	—	—	—
7–9	2.19 (0.86–5.57)	0.098	—	—
10–12	1.43 (0.51–4.04)	0.501	—	—
“3S + f” score				
4–6	1.00 (Reference)	—	—	—
7–9	1.08 (0.59–1.99)	0.806	—	—
10–12	0.83 (0.23–3.02)	0.774	—	—
MAP score				
0–1	1.00 (Reference)	—	—	—
2–3	1.52 (0.70–3.30)	0.284	—	—
4–5	1.44 (0.67–3.10)	0.346	—	—

Abbreviations: CR, creatinine; HGB, hemoglobin; OR, odd ratio.

**FIGURE 4:**
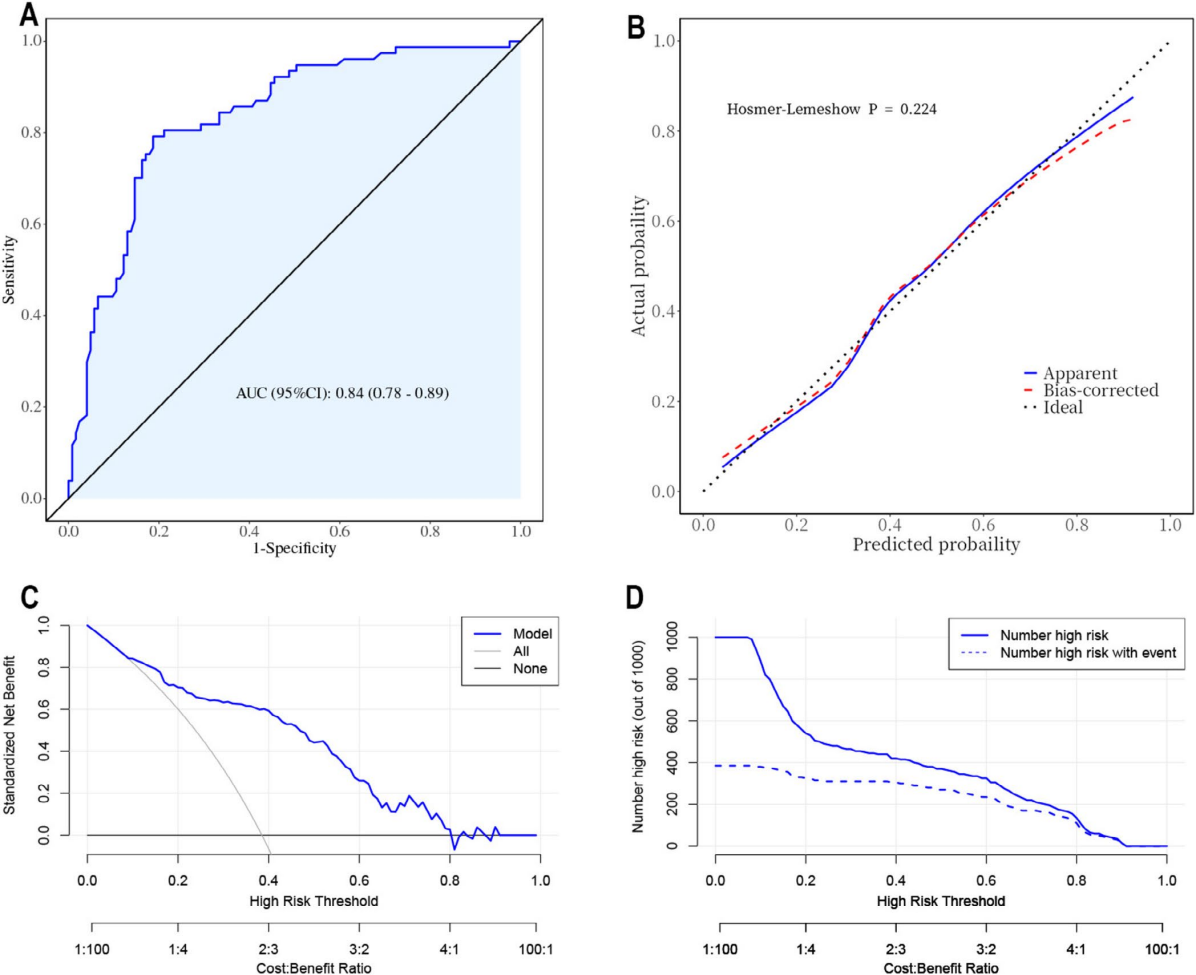
Internal validation of the prediction model. (A) ROC curve for evaluating the discriminability of the predictive model. (B) Calibration plots of the nomogram. (C) Decision curve for internal validation of the nomogram. (D) Clinical impact curve for internal validation of the nomogram. AUC, area under the curve; ROC, receiver operating characteristic.

For internal validation of the regression model, the ROC curve is shown in Figure 4A, and the area under the curve (AUC) is 0.84. The nomogram model was internally validated using a 1000 bootstrap resampling method. The calibration curve (Figure 4B) exhibited a strong consistency (Hosmer–Lemeshow *p* = 0.224) between the actual and simulated curves. In decision curve analyses (Figure 4C), it was shown that the net benefit was high, in the range from 0.1 to 0.8, suggesting benefits within a wide probability range. The clinical impact curve (Figure 4D) indicated that when the high‐risk threshold was > 80% of the predicted score probability, the nomogram determined that the approach was highly matched with the actual approach.

### 3.4 External Validation of the Prediction Model

**FIGURE 5:**
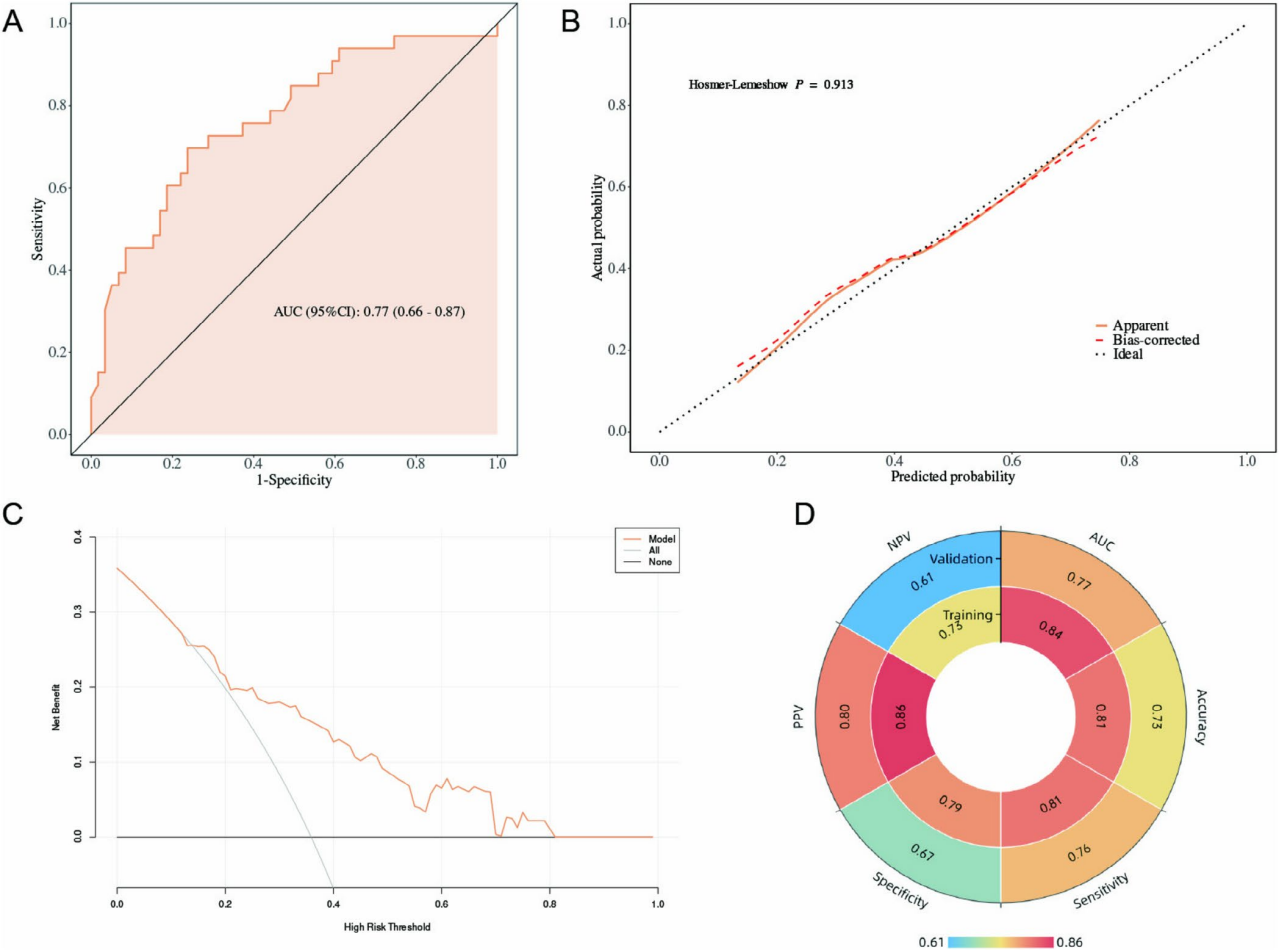
External validation of the prediction model. (A) ROC curve for evaluating the discriminability of the predictive model. (B) Calibration plots of the nomogram. (C) Decision curve for internal validation of the nomogram. (D) When using a cutoff value of 0.427 for the predicted probability, several confusion matrix‐related parameters were calculated for both the internal (inner circle) and external (outer circle) validation datasets. AUC, area under the curve; NPV, negative predictive value; PPV, positive predictive value; ROC, receiver operating characteristic.

A total of 92 patients were included in the validation cohort, with 59 cases (64.1%) undergoing the transperitoneal approach and 33 cases (35.9%) undergoing the retroperitoneal approach. We analyzed the distribution of baseline clinical characteristics between the training and validation cohorts (Table 4) and found that patients in the validation cohort had a higher prevalence of diabetes (*p* = 0.007) and a higher proportion of non‐clear cell carcinoma pathology (*p* = 0.036) and higher ASA grade (*p* = 0.020). In addition, we analyzed the differences in baseline clinical characteristics between the two surgical approach groups within the validation cohort and found no statistically significant differences in any indicators (Table 1). Data from the validation cohort were used to validate the nomogram independently. In the validation cohort, the nomogram achieved an AUC of 0.77 (Figure 5A) and the calibration curve showed moderate agreement between the predicted values and the actual observations (Figure 5B). Decision curve analysis revealed that the nomogram provided greater net clinical benefits than several alternative strategies, including intervention for all and intervention for none (Figure 5C). The confusion matrix was analyzed using the cutoff value corresponding to the maximum Youden index from the training set. The results showed that the model performed well in both the training and validation cohorts (Figure 5D).

**TABLE 4:** Comparison of baseline characteristics between training and validation sets.

Variables	Training set (*n* = 200)	Validation set (*n* = 92)	*p*
Age, years (mean [SD])	53.46 [13.01]	55.38 [13.28]	0.245
Weight, kg (mean [SD])	73.60 [13.05]	72.51 [11.79]	0.496
Hight, cm (mean [SD])	169.25 [8.14]	168.18 [8.24]	0.299
Tumor size, mm (mean [SD])	31.15 [12.14]	33.12 [12.77]	0.207
HGB, g/L (median [IQR])	148.50 [136.00, 158.00]	143.00 [134.00, 154.00]	0.050
CR, μmol/L (median [IQR])	78.50 [67.00, 88.00]	76.00 [64.75, 88.00]	0.450
Gender, *n* [%]			
Male	134 [67.00]	59 [64.13]	0.630
Female	66 [33.00]	33 [35.87]	
Hypertension, *n* [%]			
No	118 [59.00]	51 [55.43]	0.567
Yes	82 [41.00]	41 [44.57]	
Diabetes, *n* [%]			
No	167 [83.50]	64 [69.57]	0.007
Yes	33 [16.50]	28 [30.43]	
Surgical history, *n* [%]			
No	139 [69.50]	58 [63.04]	0.274
Yes	61 [30.50]	34 [36.96]	
Tumor side			
Left	102 [51.00]	44 [47.83]	0.614
Right	98 [49.00]	48 [52.17]	
Pathological type, *n* [%]			
ccRCC	165 [82.50]	66 [71.74]	0.036
nccRCC	35 [17.50]	26 [28.26]	
ASA grade, *n* [%]			
1	64 [32.00]	15 [16.30]	0.020
2	125 [62.50]	71 [77.17]	
3	11 [5.50]	6 [6.52]	

Abbreviations: CR, creatinine; HGB, hemoglobin; IQR, interquartile range; SD, standard deviation.

### 3.5 Typical Cases of Patients Underwent the Two Approaches

**FIGURE 6:**
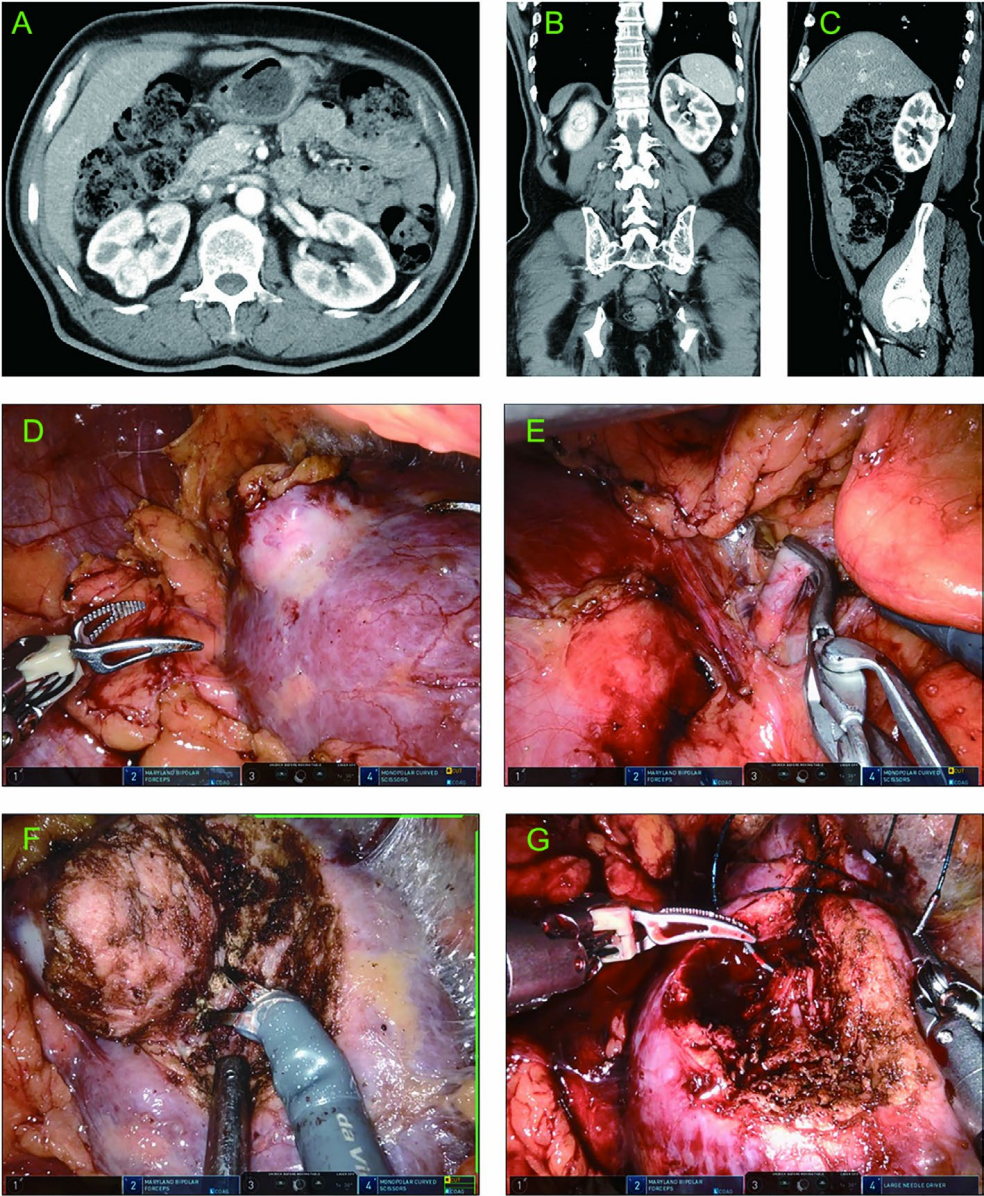
A typical case of the retroperitoneal approach. (A–C) Contrast‐enhanced CT demonstrated a rounded and enhanced‐density mass measuring approximately 2.3 cm; (A), (B), and (C) showed the horizontal, coronal, and sagittal images, respectively. (D) The tumor was well exposed in the retroperitoneal space with a clear field of view. (E) The renal artery was clamped. (F) The tumor was removed using a robotic arm without involvement of the collecting system. (G) The wound after tumor resection was sutured.

Typical Case 1: A 59‐year‐old male presented with a right‐sided renal mass during a routine health check‐up. A contrast‐enhanced CT indicated a mass of approximately 2.3 cm in diameter in the right kidney. The mass was located in the posterior and upper pole of the kidney (Figure 6A–C). According to our prediction model, the probability of RRPN was 0.85. The patient underwent RRPN (Figure 6D–G) with an arterial clamp time of 28 min, an operative time of 106 min, and an intraoperative blood loss of 5 mL. The drainage tube was removed and the patient was discharged 4 days after surgery.

**FIGURE 7:**
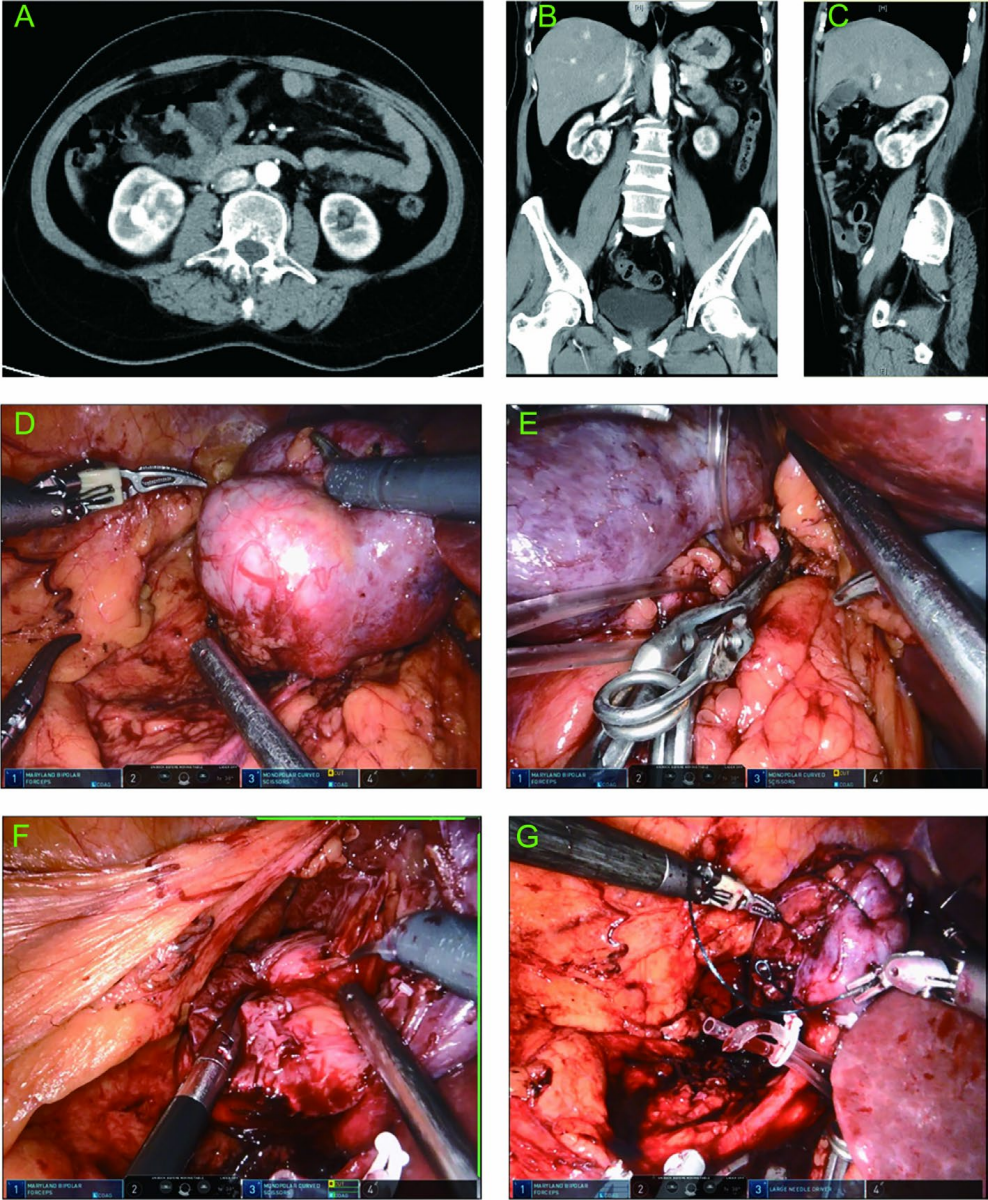
A typical case of the transperitoneal approach. (A–C) Contrast‐enhanced CT demonstrated an enhanced‐density mass measuring approximately 4.6 cm; (A), (B), and (C) showed the horizontal, coronal, and sagittal images, respectively. (D) The tumor was well exposed in the peritoneal cavity with a clear field of view. (E) The renal artery was clamped. (F) The tumor was removed using a robotic arm, and part of the collecting system was resected. (G) The wound after tumor resection was sutured.

Typical case 2: A 55‐year‐old female presented with a right‐sided renal mass during a routine health checkup. Contrast‐enhanced CT revealed a mass in the right kidney, with a diameter of about 4.6 cm. The mass was located at the anterior and lower pole of the kidney (Figure 7A–C). According to our prediction model, the probability of RRPN was 0.10. The patient underwent TRPN (Figure 7D–G) with an arterial clamping time of 25 min, an operation time of 110 min, and an intraoperative blood loss of 100 mL. The drainage tube was removed 2 days after surgery, and the postoperative hospitalization time was 6 days.

## 4 Discussion

The majority of the current research on RPN involves utilizing the transperitoneal approach [15]. This approach is favored by urologists due to its familiarity with the anatomy, adequate surgical space to avoid instrument collision [6, 16]. However, over the past decade, several reports of the retroperitoneal approach have been published, and most of them found that the perioperative outcomes were not worse than those of the transperitoneal approach [4, 7, 9, 10, 11]. Additionally, several surgeons have summarized the unique advantages of the retroperitoneal approach, such as providing better access to the posterior kidney for tumor visualization, resection, and renorrhaphy and minimizing the risk of injury to intraperitoneal organs [17, 18]. In the past, there were no clear guidelines or studies to inform surgical approach decisions. For this reason, we developed a prediction model to provide a reference for clinicians and serve as an exploratory tool for future prospective studies.

Before building a predictive model, we firstly compared the two approaches in terms of perioperative outcomes. The final results revealed no statistically significant differences for most of the variables, indicating that both approaches are comparable in terms of perioperative performance. However, we did observe significant differences in two parameters: EBL and HGB change, with the retroperitoneal approach generally showing lower blood loss and less change in hemoglobin. The findings of our study are consistent with the conclusions of a meta‐analysis published by Gu et al. [19], which also highlighted similar perioperative outcomes between the two approaches, further supporting the validity of our results.

The fact that other researchers have found that RRPN can achieve shorter WIT [10] and a reduced rate of postoperative complications [11], which are inconsistent with our findings. Additionally, some surgeons have reported shorter operative times associated with RRPN, which may be attributable to a faster access to the renal artery or the simplicity of not freeing the colon [7, 13]. In addition, our center uses the video recording tool built into the robotic system to record robotic operations and track operation time. Therefore, the time spent on trocar placement and wound closure is not included in the recorded operative time, which may result in differences in operative time compared to other studies [14]. According to Archie et al. [7] and Gin et al. [20], the hospitalization time is significantly shorter for RRPN (2.5 vs. 4.6 days, 1.5 vs. 2 days, respectively). However, in our study, there is no difference between the two groups of patients in hospitalization time. Due to the retrospective nature of the study, we reviewed postoperative complications from the written medical records, which may have resulted in some complications being omitted. This may explain the discrepancies between our study and other published research [11, 14]. In the PADORA study by Dr. Mjaess et al. [14], using a large, multicenter prospective database, the differences between RRPN and TRPN were compared. The study found that the RRPN group had lower EBL and was more suitable for posterior renal tumors, consistent with our results. It is noteworthy that they also included variables such as exophytic proportion and cystic nature, though these factors did not show statistical differences, their inclusion reflects a comprehensive study design. Furthermore, Zhang et al. categorized tumors based on location (completely upper pole, completely lower pole) and complexity (R.E.N.A.L. score ≥ 7) to examine the impact of the surgical approach [11, 21, 22, 23, 24], which inspired us to focus on tumors with specific characteristics separately in future research.

To the best of our knowledge, this is the first nomogram that predicts the surgical approach for RPN surgery. While previous studies have examined the decision of the surgical approach for laparoscopic PN, no clear preoperative determinants have been reported [23]. Liu et al. [12] researched the optimal surgical approach and concluded that the transperitoneal approach is more suitable for patients with anterior tumors and the retroperitoneal approach is more suitable for patients with posterior tumors. As can be observed, their study only took into account the tumor's anterior–posterior position. However, our model includes both demographic information and oncology characteristics.

The variables included in this model were selected using statistical methods. However, some clinically relevant variables that are often considered in actual clinical practice were not taken into account in the univariate analysis. All of the variables we included can be derived from preoperative CT scans without the need for more complex diagnostic procedures so that the nomogram can be adapted for less‐resourced settings not only for use in advanced robotic surgery environments. Even more, its underlying principles can be promoted to laparoscopic surgery.

The retroperitoneal approach for posterior tumors is clear and reasonable, as it allows for direct access to the kidney's posterior surface and renal hilum while avoiding bowel manipulation and peritoneal violation [25]. Additionally, our model shows that the tumor's location in the upper pole facilitates the retroperitoneal approach, which is consistent with a previous study [11]. The transperitoneal approach for the upper pole of renal tumors will be covered by the liver and spleen, and the difficulty of separating and suturing will be greatly increased, and the risk of liver and spleen injury may be increased during the operation [11]. In regards to tumor size, our findings indicate that larger tumors decrease the probability of adopting the retroperitoneal approach. This outcome may be attributed to the bigger space in the peritoneal cavity, which allows adequate mobilization and exposure when dealing with large tumors and effectively prevents collision of the robotic arm. In addition, our study found that the presence of accessory renal artery is usually more suitable for the retroperitoneal approach, which may be related to anatomical considerations, vascular protection, and postoperative recovery. The accessory renal arteries are usually located at the renal hilum or behind the kidney [26]. The retroperitoneal approach usually provides better protection for the kidney and its blood vessels, especially when removing tumors or renal tissues. It allows the surgeon to see the course of the accessory renal artery more clearly and reduces the risks involved in the operation.

We added the R.E.N.A.L score, PADUA score, MAP score, and “3S + f” score to our data collection schedule, since we thought that the nephrometry score systems could be contributive to determining the surgical approach when this study was initially designed. However, subsequent analysis revealed that these scoring systems do not directly impact the decision of the surgical approach, although they may be able to evaluate the complexity of the tumor. Although the total score of the aforementioned scoring systems did not show a correlation with the surgical approach, certain subcategories of them were meaningful; interestingly, these subentries were parameters related to tumor size or location (Table 2). This finding indirectly supports the rationale for determining the surgical approach based on tumor characteristics.

Although PSM effectively equalized baseline characteristics between the TRPN and RRPN groups, a significant proportion of TRPN patients were not included in the matching analysis. To examine how these discordant cases might affect the results, we performed a detailed analysis of variable distribution in both groups PSM (Table 1 and Table 3). The results showed that, apart from the four variables included in the model, there were statistically significant differences in gender, preoperative creatinine, and ASA grade. These results suggest that female patients and patients with lower creatinine levels may be more suitable for the RRPN approach, while the ASA grade showed a bimodal distribution, with higher proportions of ASA grade 1 and grade 3 in the RRPN group compared to the TRPN group. To address this issue, we attempted to develop two models by directly using the training set data (without PSM): a simplified model (variables: anteroposterior location, longitudinal location, tumor size, and accessory renal artery) and a full model (simplified model + gender, creatinine + ASA grade). The results showed that the AUC of the full model improved compared to the simplified model, but only by 0.45% (Figure 1). Considering the trade‐off between the increased complexity of the model and the small improvement in AUC, we thought that adding these additional variables did not provide significant benefit.

We acknowledge that there are some limitations in this study. Firstly, the data are from one single center and small sample size, so that the findings may not be generalizable to all surgeons. Additionally, this is a retrospective study, which may not be as robust as a randomized prospective trial. Only 87.5% (77/88) of the retroperitoneal cases and 53.5% (123/230) were matched in the PSM study, and we are unsure if there are any further unidentified or uncontrolled confounders, especially residual confounding factors related to the choice of the surgical approach may have a direct impact on the prognosis. Another crucial note is that we did not analyze the competence of the eight surgeons for heterogeneity or learning curve, which may have resulted in selection bias. Perhaps future studies will require a prospective design, randomized patient grouping, a larger sample size, and data from multiple centers to further validate our findings.

## 5 Conclusions

There are not significant differences in perioperative outcomes between the retroperitoneal and transperitoneal approaches. The retroperitoneal approach is recommended for younger patients, smaller tumors, tumors located in the upper pole, and tumors located posteriorly. Our predictive model that based on demographic information and tumor characteristics can serve as a reference for surgeon's clinical decision of the surgical approach.

## Author Contributions

Fan Shu: conceptualization; methodology; investigation; writing – original draft. Zhuo Liu: investigation; writing – original draft; writing – review and editing. Peichen Duan: methodology; writing – review and editing. Yichang Hao: data curation; formal analysis. Xin Ma: data curation; formal analysis. Hongxian Zhang: data curation; formal analysis. Guoliang Wang: formal analysis; supervision. Xiaojun Tian: formal analysis; supervision. Lei Liu: formal analysis; supervision. Shudong Zhang: conceptualization; funding acquisition; writing – review and editing.

## Ethics Statement

The studies involving human participants were reviewed and approved by the Institutional Ethics Committee of Peking University Third Hospital.

## Consent

The patients/participants provided their written informed consent to participate in this study.

## Conflicts of Interest

The authors declare no conflicts of interest.

## Supporting information


Data S1.

## Data Availability

The datasets used and/or analyzed during the current study are available from the corresponding author on reasonable request.
