# Supplementary material for: A Predictive Model for Surgical Approach Selection in Robotic Partial Nephrectomy and Its Perioperative Outcomes Based on Single‐Center Retrospective Data
Source: Cancer Med. 2025 Feb 11;14(3):e70625. doi: 10.1002/cam4.70625 (PMC11811885; doi:10.1002/cam4.70625)
Supplement: Supplementary file 1 — Data S1. [file CAM4-14-e70625-s001.docx]

**Supplementary Table 1. The differences of scoring systems and their subentry between different approach.**

| **Variables** | **TRPN (n = 59)** | **RRPN (n = 33)** | ***P*** |
| --- | --- | --- | --- |
| Age, years (mean [SD]) | 56.86 ± 13.31 | 58.30 ± 13.37 | 0.621 |
| Weight, kg (mean [SD]) | 71.30 ± 11.96 | 74.69 ± 11.31 | 0.187 |
| Hight, cm (mean [SD]) | 167.61 ± 8.74 | 169.21 ± 7.27 | 0.374 |
| Tumor size, mm (mean [SD]) | 31.78 ± 13.14 | 35.52 ± 11.90 | 0.180 |
| HGB, g/L (median [IQR]) | 142.00 [128.50, 154.50] | 144.00 [136.00, 153.00] | 0.717 |
| CR, μmol/L (median [IQR]) | 73.00 [63.00, 88.00] | 77.00 [69.00, 82.00] | 0.932 |
| Gender, n [%] |  |  | 0.405 |
| Male | 36 [61.02] | 23 [69.70] |  |
| Female | 23 [38.98] | 10 [30.30] |  |
| Hypertension, n [%] |  |  | 0.757 |
| No | 32 [54.24] | 19 [57.58] |  |
| Yes | 27 [45.76] | 14 [42.42] |  |
| Diabetes, n [%] |  |  | 0.355 |
| No | 43 [72.88] | 21 [63.64] |  |
| Yes | 16 [27.12] | 12 [36.36] |  |
| Surgical history, n [%] |  |  | 0.930 |
| No | 37 [62.71] | 21 [63.64] |  |
| Yes | 22 [37.29] | 12 [36.36] |  |
| Tumor side |  |  | 0.438 |
| Left | 30 [50.85] | 14 [42.42] |  |
| Right | 29 [49.15] | 19 [57.58] |  |
| Pathological type, n [%] |  |  | 0.875 |
| ccRCC | 42 [71.19] | 24 [72.73] |  |
| nccRCC | 17 [28.81] | 9 [27.27] |  |
| ASA grade, n [%] |  |  | 0.683 |
| 1 | 10 [16.95] | 5 [15.15] |  |
| 2 | 44 [74.58] | 27 [81.82] |  |
| 3 | 5 [8.47] | 1 [3.03] |  |

HGB, Hemoglobin; CR, Creatinine; IQR, Interquartile range; SD, Standard deviation.

**Supplementary Table 2. The differences of scoring systems and their subentry between different approach.**

| **Variable** | | **TRPN**  **(n = 123)** | **RRPN**  **(n = 77)** | **P** |
| --- | --- | --- | --- | --- |
| **“3S+f”** | Size | 1.14 ± 0.37 | 1.00 ± 0.00 | <.001 |
|  | Site | 1.85 ± 0.76 | 1.91 ± 0.79 | 0.678 |
|  | Side | 2.38 ± 0.83 | 2.15 ± 0.86 | 0.135 |
|  | Fat | 1.81 ± 0.75 | 1.85 ± 0.74 | 0.787 |
|  | Total score | 7.19 ± 1.51 | 6.91 ± 1.38 | 0.321 |
| **R.E.N.A.L.** | Radius | 1.31 ± 0.50 | 1.12 ± 0.33 | 0.007 |
|  | Exophytic/endophytic | 1.59 ± 0.69 | 1.71 ± 0.72 | 0.365 |
|  | Nearness to collecting system or sinus | 2.25 ± 0.84 | 1.94 ± 0.89 | 0.053 |
|  | Anterior/posterior |  |  | <.001 |
|  | Anterior | 67 (41.36) | 5 (14.71) |  |
|  | Posterior | 43 (26.54) | 21 (61.76) |  |
|  | Neither | 52 (32.10) | 8 (23.53) |  |
|  | Location relative to polar lines | 2.00 ± 0.83 | 1.97 ± 0.76 | 0.849 |
|  | Total score | 7.15 ± 1.81 | 6.74 ± 1.75 | 0.226 |
| **PADUA** | Longitudinal classification of the tumors | 1.36 ± 0.48 | 1.47 ± 0.51 | 0.220 |
|  | Margin location of the tumors | 1.31 ± 0.46 | 1.26 ± 0.45 | 0.614 |
|  | Relationship with renal sinus | 1.36 ± 0.48 | 1.24 ± 0.43 | 0.151 |
|  | Relationship with collecting system | 1.56 ± 0.50 | 1.47 ± 0.51 | 0.334 |
|  | Tumor deepening into the parenchyma | 1.59 ± 0.69 | 1.71 ± 0.72 | 0.365 |
|  | Tumor size classification | 1.31 ± 0.50 | 1.12 ± 0.33 | 0.007 |
|  | Total score | 8.49 ± 1.86 | 8.26 ± 1.88 | 0.526 |
| **MAP** | Thickness of adherent perinephric fat | 0.81 ± 0.77 | 0.91 ± 0.75 | 0.502 |
|  | Straining of adherent perinephric fat | 0.79 ± 1.11 | 0.76 ± 1.16 | 0.904 |
|  | Total score | 1.60 ± 1.65 | 1.68 ± 1.63 | 0.818 |

Scores, originally ordered multinomial variables, are treated as continuous variables and are expressed as means ± standard deviations. Categorical variables are expressed as frequencies (percentages). RRPN, Retroperitoneal robotic partial nephrectomy; TRPN, Transperitoneal robotic partial nephrectomy.

**Supplementary Table 3. The differences of Baseline Characteristics Between Transperitoneal and Transperitoneal Approach Before PSM**

| **Variables** | **TRPN (n = 230)** | **RRPN (n = 88)** | **p-value** |
| --- | --- | --- | --- |
|  |  |  |  |
| Tumor size, mm (mean [SD]) | 33.39 [13.02] | 27.69 [9.97] | <.001 |
| Tumor side, n [%] |  |  | 0.226 |
| Left | 108 [46.96] | 48 [54.55] |  |
| Right | 122 [53.04] | 40 [45.45] |  |
| Accessory renal artery, n [%] |  |  | <.001 |
| No | 215 [93.48] | 38 [43.18] |  |
| Yes | 15 [6.52] | 50 [56.82] |  |
| Anteroposterior location, n [%] |  |  | <.001 |
| 1 | 133 [57.83] | 20 [22.73] |  |
| 2 | 57 [24.78] | 17 [19.32] |  |
| 3 | 40 [17.39] | 51 [57.95] |  |
| Longitudinal location, n [%] |  |  | 0.002 |
| 1 | 87 [37.83] | 17 [19.32] |  |
| 2 | 81 [35.22] | 33 [37.50] |  |
| 3 | 62 [26.96] | 38 [43.18] |  |
| R.E.N.A.L. score, n [%] |  |  | 0.329 |
| 4-6 | 91 [39.57] | 36 [40.91] |  |
| 7-9 | 110 [47.83] | 46 [52.27] |  |
| 10-12 | 29 [12.61] | 6 [6.82] |  |
| PADUA score, n [%] |  |  | 0.139 |
| 4-6 | 37 [16.09] | 10 [11.36] |  |
| 7-9 | 126 [54.78] | 59 [67.05] |  |
| 10-12 | 67 [29.13] | 19 [21.59] |  |
| "3S+f" score, n [%] |  |  | 0.473 |
| 4-6 | 71 [30.87] | 33 [37.50] |  |
| 7-9 | 144 [62.61] | 51 [57.95] |  |
| 10-12 | 15 [6.52] | 4 [4.55] |  |
| MAP score, n [%] |  |  | 0.818 |
| 0-1 | 144 [62.61] | 53 [60.23] |  |
| 2-3 | 46 [20.00] | 17 [19.32] |  |
| 4-5 | 40 [17.39] | 18 [20.45] |  |

PSM, Propensity score matching; RRPN, Retroperitoneal robotic partial nephrectomy; TRPN, Transperitoneal robotic partial nephrectomy; SD, Standard deviation.


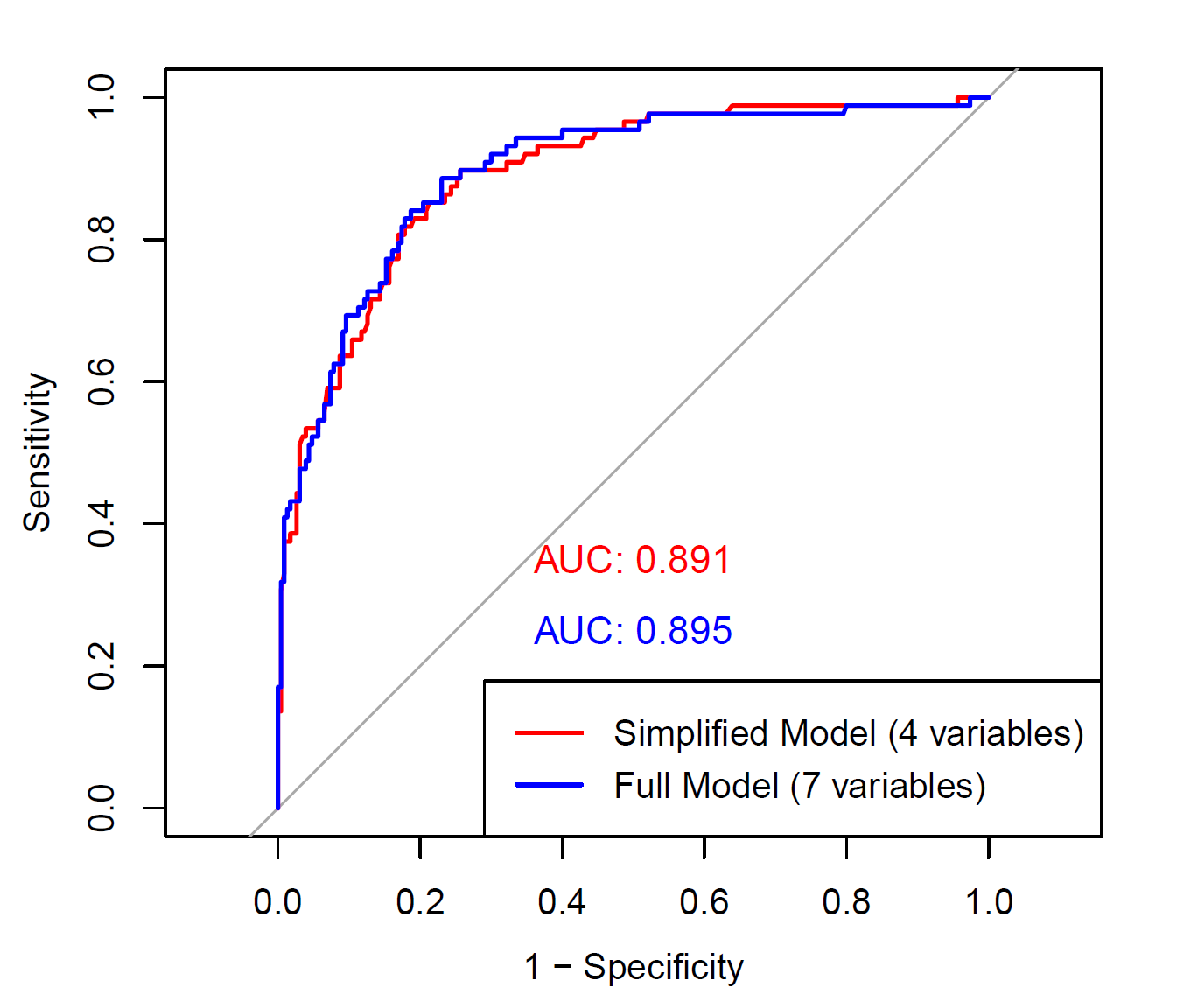


**Supplementary Figure 1. ROC curve of models based on all data in the training set.** AUC, area under curve.
